# Surgical Innovations to Protect Fertility from Oncologic Pelvic Radiation Therapy: Ovarian Transposition and Uterine Fixation

**DOI:** 10.3390/jcm13185577

**Published:** 2024-09-20

**Authors:** Ariella Yazdani, Katherine Moran Sweterlitsch, Hanna Kim, Rebecca L. Flyckt, Mindy S. Christianson

**Affiliations:** 1 Cleveland Clinic Foundation, Cleveland, OH 44195, USA; 2 Cedars-Sinai Medical Center, Los Angeles, CA 90048, USA; 3 University Hospitals Cleveland Medical Center, Cleveland, OH 44106, USA

**Keywords:** fertility preservation, ovarian transposition, oophoropexy, uterine transposition

## Abstract

As oncologic therapy continues to advance, survivorship care has widened the realm of possibilities for quality-of-life improvements, including fertility preservation and restoration. We aim to summarize the current and future directions of fertility preservation techniques for patients facing gonadotoxic medical therapies who desire pregnancy after their condition is treated. This review of both ovarian and uterine transposition highlights the present roles, techniques, and fertility outcomes of the two fertility preservation treatment modalities designed to protect reproductive organs from harmful pelvic radiation. Current evidence shows that ovarian transposition preserves ovarian function for patients with localized pelvic radiation demonstrating the most successful return of fertility. Uterine transposition holds great promise for patients desiring to conceive and carry a full-term pregnancy after radiation therapy. With ongoing advancements in oncologic treatments leading to increased survival rates, fertility is increasingly becoming a key survivorship issue. Patients can anticipate counseling about these fertility preservation surgical techniques that protect both the ovaries as well as the uterus from harmful pelvic radiation.

## 1. Introduction

As oncologic treatments continue to advance and improve survivorship, reproductive function and fertility have evolved to become a key survivorship issue for young cancer patients. While it is well known that certain chemotherapy treatments can be harmful to fertility, pelvic radiation is extremely damaging to fertility by not only depleting the oocyte supply but also damaging the uterus. Specific cancer diagnoses that frequently require pelvic radiation include colorectal cancer, pelvic lymphoma and cervical cancer [1]. This is particularly significant as new diagnoses of colorectal cancer in women of reproductive age have been increasing in recent years [2]. Over the last few decades, ovarian transposition has become a widely known fertility preservation surgical modality for young female patients undergoing cancer-related pelvic radiation therapy (RT). However, there have been few reported cases of patients with a history of pelvic radiation therapy later being able to conceive and successfully carry a pregnancy resulting in live birth. This detrimental impact on fertility is likely due to pelvic RT damaging the uterus. It is important to explore the preservation techniques that protect ovarian reserve, such as through ovarian transposition, as well as the ability to maintain the uterus to carry one’s own gestation. In this review, we aim to explore both ovarian transposition and uterine fixation as fertility preservation surgical techniques in reproductive-aged patients requiring pelvic RT and review future directions within the field of reproductive surgery for fertility preservation.

## 2. Ovarian Transposition

Ovarian transposition is an established fertility preservation technique in which the ovaries are suspended outside of the pelvic radiation field for reproductive-age patients requiring pelvic or low abdominal RT. It is often performed in young pre-menopausal patients with early-stage operable cancers of both gynecological and non-gynecological origin with the need for primary or adjuvant radiotherapy. For example, ovarian transposition has been described for colorectal carcinomas, ovarian dysgerminomas, cervical and vaginal cancers, Hodgkin lymphoma, ependymomas and sarcomas [3,4,5,6,7,8,9,10,11,12,13].

### 2.1. Role of Ovarian Transposition in Preserving Fertility

It has been demonstrated that radiation decreases ovarian follicular stores and induces ovarian atrophy through direct ionizing DNA damage [14]. The degree of damage depends on the patient’s age and dose of radiation. As such, older patients are more susceptible to ovarian damage than younger patients, due to the depletion of ovarian reserve [15]. Regardless of a patient’s age, the ovaries are extremely radiosensitive. For example, doses of less than 2 Gy are associated with up to 50% oocyte damage, and doses greater than 14 Gy result in ovarian failure [15]. This is important because most treatment plans for pelvic RT are typically prescribed at doses of 45 Gy or more [14]. Ovarian transposition is a method in which the ovaries are surgically fixed outside of the radiation field, often above the pelvic brim and as lateral as possible, decreasing damage from pelvic radiotherapy.

One of the earliest documented cases of ovarian preservation prior to pelvic RT by means of surgical suspension was described by Batten and Brown in 1958 [16]. This was a case of a young, eight-year-old female with a large pelvic neuroblastoma. At the time of laparotomy for tumor resection, it was planned to move the ovaries out of the radiation field and protect them with two separate walnut-shaped latex-coated 2 mm thick lead sheaths with a hole for the fallopian tube and broad ligament. Each ovary had a separate sheath that was then secured together and sutured to the anterior parietal peritoneum as high as possible. Two months after the completion of radiation treatment, a second laparotomy was performed to remove the lead sheaths. The right ovary was found to be healthy; however, the left ovary had unfortunately slipped out of its protective casing and was in poor condition. Years after her treatment, the patient was menstruating regularly indicating intact ovarian function [16]. Ultimately, this case was one of the first surgical fixation methods to protect ovarian function from radiation.

Simultaneously, between the years 1948 and 1957, a clinical trial was performed to assess the utility of ovarian preservation through transposition during cervical cancer surgical management [17]. The ovaries were intended to remain in situ while transposed as superiorly as possible in the abdomen during the time of extensive pelvic surgery. Ultimately, the authors showed that transposed ovaries maintained their gonadal function and improved overall health outcomes for women [17]. From the late 1950s onward, ovarian transposition became a more recognized and accepted surgical approach to preserve ovarian and reproductive function in female cancer patients undergoing pelvic RT.

### 2.2. Ovarian Transposition Surgical Techniques

A multidisciplinary approach to surgical planning is encouraged before ovarian transposition. If possible, the radiation oncologist should determine and outline the field of radiation before surgery so that the surgeon can then fix the ovaries to the optimal location. The site of transposition will vary on the radiation localization but also be determined by the patient’s anatomy [18]. Ovarian transposition can be performed by either laparotomy or laparoscopy, with laparoscopy now as the preferred method due to its minimally invasive nature and faster recovery time allowing for earlier initiation of the radiation treatment. Robotic-assisted laparoscopic ovarian transposition has also been described [19]. Damage is minimized with ovarian transposition but not eliminated, so patients are often encouraged to see a reproductive endocrinologist prior to surgical management for oocyte or concurrent oocyte/embryo cryopreservation.

There are variations in the surgical technique for ovarian transposition. Traditionally, the ovaries can be transposed either medially or laterally. In the former, the ovaries are fixed behind the uterus which acts as a shield from radiation. The ovarian ligaments and mesosalpinges are often spared [20]. Medial ovarian transposition was previously performed in patients with Hodgkin lymphoma requiring pelvic radiation; however, some reports show that lateral transposition has overall better outcomes [10,21,22]. For the lateral approach, typically, the utero-ovarian ligament is cauterized and transected, providing mobility of the ovary. The mesosalpinx and mesovarium are then dissected, and the ovary is mobilized with its blood supply skeletonized. The ovary can then be sutured to the ipsilateral anterior-lateral abdominal wall, above the pelvic radiation field with its blood supply intact. The ovaries are secured to the peritoneum at either two or three points to avoid torsion. Radio-opaque clips are then often applied for identification in future imaging in case of unforeseen ovarian migration [9,18]. With this approach, the fallopian tubes may remain intact to maintain the possibility of spontaneous pregnancy or may be transected. If removed, the patient will then require in vitro fertilization for oocyte retrieval and embryo implantation. Of note, it is important to consider the cancer diagnosis when deciding to maintain the fallopian tubes or not [23,24].

New approaches and surgical techniques are constantly being described for safe and effective ways for ovarian transposition. It can involve moving one or both ovaries at least 3 cm from the upper border of the radiation field, as this has been shown to preserve ovarian function [25]. Through a retroperitoneal tunnel, the ovaries or adnexa may be positioned as high as possible at the paracolic gutters while maintaining blood supply. Often, absorbable sutures are used to fix the ovaries to the peritoneum. With this technique, there is the advantage of spontaneous ovarian repositioning but also the risk of migration of the ovaries back to the radiation field before the end of treatment [20]. Another technique, namely, percutaneous needle transposition, described by Gareer et al., allowed for a simpler, easy-to-perform approach to ovarian transposition [26]. This technique involves laparoscopically cutting the utero-ovarian ligament and fixing the ovaries to the anterior abdominal wall by introducing a percutaneous straight needle through a 2 mm skin incision at the site of fixation. This allowed for outpatient repositioning of the ovaries by simply cutting the subcutaneous suture using a local anesthetic, avoiding a second operation [26]. This technique, however, is limited to patients undergoing a short radiation period. Lastly, as early as 1968, there were reports of exteriorization of the ovary to subcutaneous fat tissue [27]. In this method, the ovarian vessels were sutured to the peritoneal surface, increasing the risk of adhesion formation and possible intestinal herniation. In 1996, a modified technique of subcutaneous transposition using the retroperitoneal space as a route was described by Fujiwara et al. [28]. Similar to previously described techniques, the utero-ovarian ligament is dissected, and the ovarian pedicle is dissected off of the peritoneum. The retroperitoneal space is widely opened, and the skin and fascia where the ovary is transposed is incised, allowing for fixation on the fascia before closure of the skin. This technique would allow for the added advantage of early diagnosis of ovarian cysts, easy access to remove those cysts as well as a simple approach to the ovaries for future in vitro fertilization [28].

Furthermore, a combined approach in which a unilateral ovary is transposed with concomitant cryopreservation has been reported [12,13,29,30]. With this strategy, one ovary would either be removed or undergo a biopsy for ovarian tissue cryopreservation. The other ovary would then be transposed by the means described above. Combining ovarian preservation approaches, such as ovarian transposition with cryopreservation, is thought to maximize the chances of future fertility. It is important to consider a combined approach and offer this technique to all reproductive-aged patients before the initiation of pelvic radiation.

### 2.3. Ovarian Transposition Reported Outcomes

Despite being first described in the late 1950s, there are variable documented pregnancy and birth rates after ovarian transposition [17]. Success is influenced by many factors such as the age of the patient, dose of radiotherapy, distance of the transposed ovary from the radiation field, and the need for concurrent chemotherapy [11,13].

One meta-analysis of 24 studies looked specifically at outcomes of ovarian transposition in gynecologic cancers in 892 patients [8]. In the brachytherapy alone group, 94% (95% CI 79–111) of patients had preserved ovarian function with transposition. Ovarian function was defined by the patient’s symptoms and serum follicle-stimulating hormone, luteinizing hormone, and estradiol levels. In patients who underwent ovarian transposition followed by subsequent external beam radiotherapy (EBRT), with or without brachytherapy, the proportion of patients with preserved ovarian function decreased to 65% (95% CI 56–74). Each group had a minimal risk of developing cysts or metastases to the transposed ovaries [8]. Another systematic review of 38 studies with 765 patients showed that ovarian survival was greatest after ovarian transposition and brachytherapy (63.6–100% survival) when compared to ovarian transposition and EBRT (20–100% survival), with a low complication rate (0–28.6%) [31]. Complications were defined as ovarian cyst development, abdominal pain, hematoma, unintended tubal ligation and ischemia. These data are important when counseling young patients on the risks to fertility with EBRT when compared to brachytherapy. A more recent systematic review that looked at ovarian transposition in over 1350 patients with cervical cancer showed that ovarian function was preserved after pelvic radiotherapy (brachytherapy and/or EBRT) with or without chemotherapy (61.7%, with a range of 16.6–100%) [7]. Not surprisingly, the distance from the radiation field is the factor most significantly reported to be correlated with perseveration of ovarian function after transposition [32]. Even with ovarian transposition and a lead block to shield reproductive ovaries during RT, the ovaries can still receive up to 8–15% of the radiation dose through scatter radiation [33]. These variations in success rates reinforce the many factors that may influence ovarian function after radiotherapy and must not be overlooked.

It is important to also comment on complications that may arise from ovarian transposition, although these are rare. A meta-analysis by Buonomo et al. reported 117 complications after a review of 28 studies, including a total of 1377 patients with cervical cancer who underwent ovarian transposition surgery [7]. The most commonly reported adverse outcomes were ovarian cysts (94.9%), abdominal pain (3.4%), as well as a small risk of small bowel obstruction due to post-surgical adhesions (0.85%) [7]. Further complications may include ovarian torsion, infarction of fallopian tube, chronic pelvic pain and migration of the ovaries back into the radiation field [9,14,22,24,34]. Oophorectomy at the time of transposition is an unintended, yet rare, complication either due to damage of the gonadal vascular supply at the time of the primary procedure or due to secondary causes such as large cysts or torsion necessitating removal [24]. Additionally, as with any abdominal surgery, pelvic adhesions may form leading to fallopian tube occlusion, increasing infection risk and decreasing fertility or, as previously mentioned, bowel obstruction [35]. Besides these unfortunate yet benign complications of transposition, it is important to review the sequelae associated with cancer. Complications of ovarian transposition in association with the primary cancer diagnosis may include incomplete surgical staging, subsequent recurrence in the ovary or ectopic metastasis at the abdominal wall [24,34,36,37,38,39]. The in situ transposed ovaries could be potential sites for metastasis of the primary cancer, especially for cancers known to spread to the ovaries such as sarcomas or cervical cancer [36,40,41]. The data regarding the risk of cancer recurrence from transposed ovaries are limited, but it has been documented to occur 2 to 8 years later for non-gynecologic cancers [34,40,42]. Additionally, there have been case reports of abdominal wall metastasis near laparoscopic trocar sites, yet this may be more attributable to the stage of cancer at the time of surgery [37]. A review published in 2013 showed 7% of cryopreserved ovarian tissue had malignant infiltration [36,41]. Therefore, when selecting candidates for ovarian transposition, it is important to consider the current extent of disease, as well as establish better guidelines for surveillance in individuals who undergo transposition [38,39].

## 3. Uterine Fixation

Uterine transposition, or fixation, is a fertility-sparing surgery that aims to protect the uterus, mainly the fundus, from radiotherapy damage while allowing for continued menstruation. While there is increased retention of ovarian reserve with techniques like ovarian transposition as outlined in previous sections, the ability to carry a pregnancy after pelvic radiation is limited due to uterine damage. This newer procedure was first described in 2017, many years after the first ovarian transposition, for a patient with rectal adenocarcinoma [43]. However, there is increasing research on the efficacy of uterine transposition as a fertility preservation modality for the uterus to successfully allow pregnancy after pelvic radiation treatment [44,45].

### 3.1. Role of Uterine Fixation in Preserving Fertility

The damaging effects of RT on the uterine environment has been studied for decades. For example, an irreversible effect on uterine development (i.e., uterine length and elasticity) and uterine vasculature has been shown in observational studies of childhood cancers treated with abdominal and pelvic radiotherapy [46,47]. Further, RT can impair uterine distensibility due to myometrial fibrosis and abdominal pelvic fibrosis and cause direct endometrial injury which, in turn, can increase the risk for pregnancy-related complications including spontaneous miscarriages, preterm deliveries and placental abnormalities [46,48,49,50,51,52]. The risk of uterine damage is dependent on the radiation dose, site of RT, and patient age, with the pre-pubertal uterus being more vulnerable to pelvic radiation [47,48].

Recognition of the fact that radiation damage to the uterus was preventing women with a history of pelvic radiation to successfully carry pregnancies has guided researchers to develop innovative ways to protect the uterine environment. Ribeiro et al. described the first reported case of uterine fixation in 2017 in a patient requiring pelvic radiation in the setting of rectal adenocarcinoma [43]. Soon after, in 2018, Baiocchi et al. described the first case of uterine transposition after radical trachelectomy [53]. Then, Marques et al. in 2020 reported the first robotic-assisted laparoscopic case of uterine transposition [54]. In 2021, the first reported case of uterine transposition was performed in a three-year-old pediatric patient [55].

### 3.2. Uterine Fixation Surgical Techniques

As previously mentioned, Ribeiro et al. described the first case of successful transposition of the uterus outside of the radiation field as a means for fertility preservation [43]. This was a case of a 26-year-old patient diagnosed with rectal adenocarcinoma. By means of laparoscopy, the surgeons completely mobilized the uterus, creating a colpotomy and dissecting the infundibulopelvic ligaments up to the intersection of the iliac vessels. The uterus was then positioned in the upper abdomen, with the round ligaments and gonadal vessels directly sutured to the anterior abdominal wall, with the goal of keeping the uterus away from the radiation field. The cervix was then fixated to the umbilicus and anastomosed directly to the fascia, allowing for menstruation throughout treatment and visual inspection of the cervix. Five weeks after radiotherapy, the uterus was then repositioned and re-implanted in the pelvis, and menstruation resumed two weeks later [43]. Similarly, this formation of a cervical stoma to allow for menstruation was successfully reported in a separate 36-year-old patient with moderately differentiated rectal adenocarcinoma [56].

Following the first uterine transposition, in 2018, Baiocchi et al. described the first case of uterine transposition for fertility sparing in a patient with cervical cancer [43,53]. This was a case of a 33-year-old patient who had a radical trachelectomy in the setting of stage Ib1 cervical cancer that required adjuvant radiotherapy. Through laparoscopy, the uterine corpus and ovaries were detached from the vaginal anastomoses from the prior trachelectomy, mobilized and sutured to the right upper abdominal wall remote from the EBRT site. One week after radiation, the uterus and ovaries were repositioned, and six months later, the patient had regular menses with no evidence of recurrence [53].

The first documented robotic-assisted uterine transposition procedure was performed in a 28-year-old woman with squamous cell carcinoma of the cervix and lymph node micro-metastasis who refused adjuvant radiotherapy because of the risk of infertility [54]. During this procedure, the uterine corpus and ovaries were detached from the pelvis and robotically sutured to the anterior upper abdominal wall. In this case, the short residual cervix from prior procedures did not allow for umbilical implantation. Ten days after transposition, external radiotherapy was delivered. After treatment completion, the uterus and ovaries were robotically repositioned, and the cervix was re-anastomosed to the vagina. Twenty months later, the patient regained normal menses and hormonal function [54]. This case demonstrates that robotic-assisted uterine transposition may represent a separate minimally invasive approach to laparoscopy.

Overall, these cases demonstrate distinct minimally invasive surgical methods of uterine transposition. The initial radical approach showed a successful way for uterine transposition in the setting of rectal adenocarcinoma in which the uterus is fully mobilized with cervical stoma formation at the site of the umbilicus [43,56]. The cervical cancer case for which the patient successfully underwent uterine mobilization and suspension without formation of a cervical stoma displayed advances via a robotic approach [53]. Both novel approaches show innovation and the growing possibilities for fertility-sparing surgery.

Furthermore, it is worth mentioning uterine ventrofixation as another uterine displacement technique for fertility sparing in cancer patients. In brief, uterine ventrofixation is a suspension technique that may be obtained by using the round ligaments or by directly fixating the fundus to the anterior abdominal wall. Unlike uterine transposition, the ovaries must first be transposed before uterine ventrofixation. One case report described a laparoscopic procedure in which the uterus was fixed with three resorbable sutures into the fascia of the anterior abdominal wall as cranial as possible [57]. This method is noted to be a feasible and effective means of decreasing the effects of radiotherapy on the uterus.

### 3.3. Uterine Fixation Outcomes to Date

Unlike ovarian transposition, there is a paucity of research on the efficacy of uterine fixation as a surgical approach to fertility preservation. As already described, there is a limited number of case reports documented on the use of uterine transposition. One prospective multicenter observational study looked at uterine transposition as a means of fertility preservation in patients with non-gynecologic pelvic cancers who underwent pelvic radiation [58]. Eight patients were included, seven with rectal cancer and one with pelvic liposarcoma, who underwent uterine transposition. The uterus was preserved in six (75%) patients with three of these patients going on to attempt to achieve pregnancy. However, one patient died of carcinomatosis after uterine transposition, while another presented with uterine necrosis four days after uterine transposition necessitating removal. Of the three patients who attempted to become pregnant, two (66%) spontaneously conceived and delivered at 36 and 38 weeks by cesarean section [58]. This study shows that uterine transposition surgery can preserve the ability of young cancer patients to conceive and carry full-term pregnancies.

Furthermore, preliminary results of a systematic review on uterine displacement techniques (i.e., ventrofixation vs. transposition) showed that uterine transposition, as described by Ribiero et al., was the most protective from RT. However, the analysis concluded that ventrofixation of the fundus could still be considered as a fertility-sparing approach in young rectal and anal cancer patients [43,59].

## 4. Conclusions

With ongoing advancements in cancer treatment, survival rates have drastically improved, shifting the focus to considering important aspects of quality of life, particularly fertility. Pregnancy following pelvic radiation is rare and poorly documented in the literature. This is in part due to pelvic radiotherapy damage to the reproductive organs. While assisted reproductive technology (i.e., oocyte/embryo cryopreservation, in vitro fertilization) or gestational surrogacy programs are more colloquially understood and accepted, they may not always be the most accessible options. Although the use of fertility preservation services in female reproductive-aged cancer patients is undoubtedly increasing, the rate of referral to services remains low. It has been shown that clinical, socioeconomic and regional disparities may influence access to fertility preservation services [60]. Advancements in surgical fertility-sparing methods could be an answer to a more equitable future, and thus, special attention must be directed towards holistically inclusive patient referrals.

### 4.1. Future Directions

While ovarian transposition has become an established method of fertility-sparing surgery, uterine transposition is still in its early stages and is currently investigational. Even so, ovarian transposition has variable success rates and is still largely underutilized. This is likely multifactorial due to factors such as lack of awareness by both patients and providers, lack of surgeons who perform the procedure, and lack of information about safety and future complications [61,62]. We know, however, that there is a need to further establish uterine transposition as a means for fertility-sparing surgery. Despite the variable success rates of ovarian transposition and the overall lack of research on uterine transposition, we argue that there is enough convincing evidence to better study and further promote both fertility preservation techniques.

A way to utilize these techniques would be for institutions with specialized surgical groups to establish protocols that would help educate and train providers on ovarian and uterine transposition. This would then allow providers to initiate comprehensive discussions on the risks and benefits of both surgical options with their patients, allowing for an informed, shared decision regarding reproductive options. These groups may also promote more opportunities for research development towards improvements in the field of reproductive surgery.

### 4.2. Special Considerations

Understanding that ovarian and uterine transposition are novel approaches to fertility-sparing techniques, there are some special considerations worthy of mention. For example, we must keep in mind patient-centered and patient-specific care when recommending such surgical interventions. Many fertility-preserving surgeries are still not covered by insurance and thus are costly for families. Further, fertility preservation must not delay the onset of any cancer-related treatment. Surgical complications of ovarian transposition and uterine transposition, albeit limited in the current literature, might arguably delay the onset of treatment therapy, at least in the early stages of development. As noted, these surgeries are technically complex and require skilled surgeons. There is opportunity to expand access to ovarian transposition and uterine fixation as a fertility preservation option as more surgeons are trained and research advances. Lastly, there is an important need for an interdisciplinary approach to refining these surgical techniques which will require engagement from multiple subspecialists including, but not limited to, surgical oncology, radiology oncology and reproductive endocrinology.

## Data Availability

No new data were created or analyzed in this study.
